# Swill Milk

**Published:** 1858-07

**Authors:** 


### EDITORIAL DEPARTMENT.

---

*Swill Milk. Is it Poisonous?*—The terrible picture of the swill milk trade in New York, so graphically drawn by Frank Leslie, seems to have opened the eyes of the people to some of the abuses of which they are the victims; and to have directed their attention, temporarily, we fear, to the preservation of health in large cities. We fear that the illustrations which we have seen, of the housing and feeding of cows, by those who are extensively engaged in the milk trade, are little, if at all, exaggerated; but we are rather inclined to believe that these excessive abuses are confined to the city of New York. The developments which have recently been made are by no means new; our transatlantic brethren, whom we can always trust for giving us the full credit of anything of such a nature, have long since been acquainted with this feature of New York dietetics. Hassall on "Food and its Adulterations," mentions an account in the Veterinary Record, copied from a New York paper, of the state of the milk stables in the vicinity of that city. He gives nearly the same account as that which has lately produced such an excitement. The cattle he describes as crowded as many as possible into the most filthy dens, with no air, no exercise, no diet but the reeking refuse of the distillery, which produces a most revolting condition of the poor victim, who never goes out alive. The symptoms induced by this mode of treatment are described in nearly the same words. The gland is described as being unnaturally stimulated, secreting a larger quantity of milk, of a much inferior quality, which does not diminish as the health of the animal fails, but of course partakes of the general disease. All this was published as early as 1850, yet the evil has continued undisturbed up to the present time.

In reviewiug the late developments in this matter, we can easily see two great causes of the disease which prevails in the New York cow stables. First, the confinement of such great numbers in a limited space, want of air exercise, &c., which we can conceive sufficient to produce these effects even with the best diet; and second, the restriction of the animal tc almost a single element of food. It is unnecessary to discuss the first proposition. Every one is acquainted with the effects of such a procedure. A cow cannot live in such an atmosphere, so crowded as hardly to be able to lie down, any more than the wretched prisoners in the famous black hole of Calcutta. The second proposition has lately been abundantly proved by the experiments of Dr. Hammond, U. S. A., who has demonstrated that life cannot be sustained by a single element, though it be of the most nutritious character; and when we look a little into the chemistry of the distillery refuse, we will see, that although it is possessed of an element which is very nutritious, yet this is almost its only constituent. These two causes seem to us to be sufficient to produce all the revolting consequences which have lately been brought to light.

For the purpose of being able to speak somewhat advisedly about the properties of the swill and the swill milk, as we find it in our own city, we procured a quantity of the feed, and several samples of milk, which we examined as carefully as our limited stock of office chemicals would permit. We even went so far as to taste the feed, which, by the way, is by no means disagreeable. It has a sweetish peculiar taste of which cattle are excessively fond. It is of a whitish color and of about the consistence of milk. On being allowed to stand for an hour or so, it deposits an abundant sediment, composed of the *debris* of the grain. Its reäction is decidedly acid. There is no alcoholic taste, and in examining the processes for the separation of the alcohol, it is evident that if there be any remaining, it must be in quantity so minute that it may be disregarded. Inasmuch as the object of distillation is to convert the starch into sugar and thence into alcohol, we did not expect to see the decided reäction which followed the addition of a solution of iodine to the liquid. A small quantity diluted with thirty or forty times its bulk of water struck a decided blue with iodine, showing that some of the starch was still unchanged. Trommer's test detected the presence of a very minute quantity of sugar.

We see from this rough analysis, that nearly all the starch is converted into sugar, and nearly all the sugar into alcohol; both the iodine test for starch, and Trommer's test for sugar, being exceedingly delicate, and capable of detecting very minute quantities of either of these substances. Then, of the important elements, we have the gluten and cellulose only remaining in quantity. Gluten is exceedingly nutritious, being in fact, the vegetable fibrin, and the most important element in the nutrition of the muscular system, but when not combined with a greater quantity of other matters than in the substance under consideration, it is utterly inadequate to the support of life. This fact has been established beyond question by numerous experiments. As far as chemistry goes, therefore, we can see nothing *absolutely injurious* in the distillery refuse, though when animals are exclusively confined to it, it will produce the most serious effects upon the health and necessarily upon the quality of the milk.

There are few cows in this city and the vicinity, however, which are thus fed; the majority of them are fed as usual, upon grass, hay, or mill-feed, and given twice or three times a day, as much of this compound as they will drink, in the place of pure water. This has no bad effect upon the health, the animal grows fat, apparently depositing healthy fat, and gives an increased quantity of milk, though of an inferior quality. We have no doubt that nearly every family in this city are in the habit of using this milk, and we venture to say, without any bad effects, the only differences being a poverty in organic matter.

In order to make some comparison between the varieties of milk, we procured a number of specimens of morning's milk, which we examined on the same day. One of them we procured personally from a farm a few miles out of the city, and we were assured that the cow had never been fed with distillery slops, but had been out to grass. Another we procured from a cow which was constantly kept in a barn in the heart of the city, but had good feed and no distillery slops. The other specimen was from a cow which was well fed, but allowed to drink the distillery refuse twice a-day. We also procured two or three other specimens of the same kind. The country cow gave twelve quarts; the city cow, five quarts; and the cow fed on swill, sixteen quarts of milk daily, showing a most decided effect of the swill in increasing the lacteal secretion.

In examining these specimens microscopically, the first showed an immense quantity of oil globules, being exceedingly rich. The second was by no means so rich, but was still a fair specimen of milk. The third, (the swill milk,) was still poorer in globules, and the globules had a slight tendeney to agglommerate.

We then coagulated the casein by heating the milk with a few drops of acetic acid. The quantity of whey from the first specimen, the country milk, was very small. The quantity from the second specimen, the city milk, was about one-half greater. The quantity from the third specimen, the swill milk, was about twice as great. This, of course, indicated a quantity of casein, inversely proportioned to the quantity of whey.

These experiments, though very rough, are sufficient to show that the milk of cows fed with distillery slops is wanting in the fats and casein, and is consequently much less nutritious. This is the manner in which most dairymen of this city, feed their cattle. We will now for a moment glance at the apparent effect which this milk produces on the health of children and adults.

It is only for the last three years that milkmen have been able to procure this feed for their cows, and the mortality among children has not increased in that time out of proportion to the increase of population. We have never seen a well-authenticated case of disease which could be traced immediately to the consumption of ordinary milk. Cows which are fed moderately on distillery refuse, in addition to an ordinary food diet, do not suffer in health, and give a larger quantity of milk, though inferior in richness, but still, containing no poisonous principle.

Taking every thing into consideration, we believe that the disease in the cow stables of New York is due to the excessive crowding, want of exercise, and confinement to a single article of diet, which, though it is nutritious, will not alone sustain life. This evil calls for speedy removal, and cannot but have a most deleterious effect upon the health of the city.

We conceive, however, that there is nothing in our own city, which can be compared to this; there is no necessity for crowding the cows, or confining them exclusively to slops, such as exists in the city of New York. The milk that we get, then, cannot be poisonous, or injurious to ordinary persons; and although of course it is not as good as the delicious country milk, a specimen of which we examined, yet we think it is as good as we can expect to get ordinarily in a city of an hundred thousand inhabitants. We do not think that our milk is injurious even to children, if they be healthy and above a certain age. Young children, however, for whom we find a substitute for the natural nourishment, should never be fed on it, merely on account of its deficiency in nutritious matter, for it is unnecessary to tell the physician how important it is in such cases, to obtain the purest richest milk, and that from a single source. The delicate organization of the young infant is ill suited to bear a change from its natural nourishment, and we certainly would not advise to bring it up on swill milk.

The experiments which we made were necessarily very imperfect; we made no attempt at minute quantitative analyses of the slop feed, or the milk, or even in the case of the milk, to form any estimate of the relative quantities of sugar, or inorganic matter, which are seldom very varied, and not of such importance as the butter and casein. These minute investigations we leave for experienced chemists. The reader must take what we have said for what it is worth, and in giving some of our data, we enable enable him to form some estimate of the value of our opinion.

(NOTE.)—We have since examined, microscopically, several other specimens of milk from cows allowed to drink distillery slops twice or three times daily, finding it always poorer in quality, but have not observed again, any tendency in the globules to agglommerate.

